# PTPRM Is Critical for Synapse Formation Regulated by Zinc Ion

**DOI:** 10.3389/fnmol.2022.822458

**Published:** 2022-03-21

**Authors:** Xiaoqiang Mo, Mengxue Liu, Jihong Gong, Ying Mei, Huidan Chen, Huajun Mo, Xiaofei Yang, Jun Li

**Affiliations:** ^1^Affiliated Hospital of Youjiang Medical University for Nationalities, Baise, China; ^2^Youjiang Medical University for Nationalities, Baise, China; ^3^Key Laboratory of Cognitive Science, Laboratory of Membrane Ion Channels and Medicine, and College of Biomedical Engineering, South-Central Minzu University, Wuhan, China; ^4^Wuhan Institute of Biological Products, Co., Ltd., Wuhan, China

**Keywords:** zinc ion, synapse, synapse formation, synaptic transmission, synaptogenesis

## Abstract

In the nervous system, the trace metal ion zinc is required for normal mammalian brain development and physiology. Zinc homeostasis is essential for the control of physiological and pathophysiological brain functions. Synapses, the junctions between neurons, are the center of the brain’s information transmission. Zinc deficiency or excess leads to neurological disorders. However, it is still unclear whether and how zinc ion regulates synapse formation. Here, we investigated the effect of zinc on synapse formation in a cultured neuron system, and found that synapse formation and synaptic transmission were regulated by zinc ions. Finally, we identified that PTPRM is the key gene involved in synapse formation regulated by zinc ions. This study provides a new perspective to understanding the regulation of brain function by zinc ion.

## Introduction

The trace metal ion zinc, serving structural, catalytic, and regulatory roles in cellular biology, is required for normal mammalian brain development and physiology. Neurons containing “free ionic zinc” (Zn^2+^) exist in many areas of the brain, such as the hippocampus, cortex, amygdala, olfactory bulb (Bitanihirwe and Cunningham, 2009; Choi et al., 2020). Zinc ions enter the synaptic vesicles through their Zn^2+^ transporters (ZnTs), then store together with glutamate. About 10% of all Zn^2+^ distribute in the synaptic vesicles of glutamatergic neurons and is released upon the vesicle fused with the plasma membrane (Howell et al., 1984; Kitamura et al., 2006). Zinc is released along with glutamate into the synaptic cleft and acts on postsynaptic receptors, such as GABA receptor, NMDA receptor, or voltage-gated channel (Ohana et al., 2004; Bitanihirwe and Cunningham, 2009). In the nervous system, ion zinc is maintained in a proper concentration with a complex and precise regulatory mechanism. The concentration of Zn^2+^ in the cytoplasm is about the picomolar range, while rises to micromolar levels in the proximity of axon terminals following release from synaptic vesicles that contain millimolar (Frederickson et al., 2005). According to previous studies, in the synaptic cleft, peak concentrations of Zn^2+^ were in the 100–300 μM range (Assaf and Chung, 1984; Howell et al., 1984; Kitamura et al., 2006). Zinc deficiency or excess leads to neurological disorders. Zinc deficiency induces neurogenesis and cognitive dysfunction, such as increased neuronal death and decreased learning and memory (Takeda and Tamano, 2009; Adamo and Oteiza, 2010). In the 1980s, studies have shown that zinc deficiency impaired the maturation of Purkinje cells and cerebellar cortex development (Dvergsten et al., 1984). Later, it was found that embryos with reduced neural progenitor cell proliferation in the ventricular zone with the zinc-deficient diet during mother pregnancy (Nuttall et al., 2015). Then, zinc deficiency not only impairs neuronal precursor cell proliferation but also inhibited neuronal differentiation in human neuroblastoma cell (IMR-32) culture experiments (Corniola et al., 2008; Adamo et al., 2010). Furthermore, zinc deficiency alters the hippocampal gene expression and impairs neuronal differentiation (Gower-Winter et al., 2013). Increasing evidence suggests a link between zinc deficiency and depression. Clinical data have demonstrated a lower serum zinc concentration in patients with depression (Wang et al., 2018). In particular, serum zinc levels were negatively correlated with the severity of depression (Nowak et al., 2005). By contrast, antidepressant therapy causes an elevation in zinc levels (Nowak and Schlegel-Zawadzka, 1999; Nowak et al., 2003). It has been clearly supported by animal models of epilepsy that synaptic zinc has a great relationship with seizure activity (Takeda et al., 2003; Flynn et al., 2007; Foresti et al., 2008). In addition, the presence of zinc in the basolateral amygaloid terminals may play a crucial role in the GABAergic neuronal cell loss found in schizophrenia models (Lipska, 2004; Woo et al., 2008). In short, zinc deficiency affects the development and functions of the nervous system.

Excess zinc has been shown to play a role in neurotoxicity associated with traumatic brain injury, stroke, seizures, and neurodegenerative diseases. During forebrain ischemia, considerable zinc is released from synaptic vesicles and accumulated in hippocampal CA1 pyramidal neurons, leading to delayed neuronal death (Sensi et al., 2011). Studies show that the level of extracellular zinc ion reached peak value (600 nmol/L) after 15 mins of ischemia, then decreased and returned to the basal level 15 min after reperfusion. However, the pyramidal cells in the hippocampal CA1 region showed intracellular zinc accumulation 24 h after ischemia (Kitamura et al., 2006). The number of zinc positive cells was positively correlated with the ratio of cerebral infarction volume in the middle cerebral artery occlusion (MCAO) model of rats. Zinc excess causes serious mitochondrial dysfunction, calcium homeostasis disorder, and ROS (reactive oxygen species) release, leading to acute cell necrosis (Lee et al., 2006; He and Aizenman, 2010). High concentrations of zinc are observed in the amyloid deposits from both AD patients and AD-prone transgenic mice (Friedlich et al., 2004; Zhang et al., 2008). Zinc-rich amyloid plaques are likely to promote the aggregation of amyloid-b protein (Aβ), as the interactions of Aβ with zinc can lead to its aggregation *in vitro* (Dong et al., 2003). Therefore, zinc homeostasis is essential for the control of physiological and pathophysiological brain functions (Sensi et al., 2009; Paoli, 2014). However, the mechanism of zinc ions in regulating nervous system function and leading to diseases remains unclear.

Synapses, the junctions between neurons, mediate the communication of brain network information (Ovsepian, 2017; Südhof, 2018; Shin et al., 2019). Synapses are composed of presynaptic membrane, postsynaptic membrane, and the synaptic cleft. Presynaptic vesicles encapsulated neurotransmitters, fused with presynaptic membrane, released neurotransmitters to synaptic cleft, acted on postsynaptic, completing the signal transduction (Südhof, 2004). The formation and functions of the synapse are the basis for the realization of advanced behaviors such as brain cognition, memory, and thinking (Chua et al., 2010; Südhof, 2018). So far, it is still unclear whether and how zinc ion regulates synapse formation.

Here, we investigated the effect of zinc on synapse formation in the cultured neuron system, and found that synapse formation and synaptic transmission were impaired by zinc ions excess. The expression of PTPRM gene was decreased in high Zn^2+^ treated neurons. Recovery of PTPRM expression could rescued the impairments caused by Zn^2+^ excess. Our results provide a new perspective to the mechanism of zinc ion on neuronal function.

## Materials and Methods

### Cell Culture

HEK293T cells (CRL-11268, ATCC) were cultured in Dulbecco’s modified Eagle’s medium (Gibco) with 10% fetal bovine serum (Gibco) and penicillin–streptomycin (100 μg/ml and 100 μg/ml).

Cortical neurons were obtained from postnatal day 0 (P0) pups of Kunming mice, as described previously (Wang et al., 2020). Briefly, the cortex was dissected from newborn mice, digested to single cell with 0.25% trypsin (Gibco) for 12 min at 37°C. Then cells were plated at a density of 80,000 per circular glass coverslips (12 mm diameter) coated with poly-L-lysine (Sigma). Cells seeding medium contains MEM (Gibco), 2% (v/v) B27 (Gibco), 0.5% (w/v) glucose, 100 mg/L transferrin (Sigma), 5% (v/v) fetal bovine serum (Gibco), and 2 mM Ara-C (Sigma). During the process of neuron culture, the growth medium was needs to replace in DIV1 (the day 1 *in vitro*), DIV4, and DIV9. Each time 500 μl medium was taken out and 600 μl was added in. Different concentrations (0 μM, 10 μM, or 50 μM) of Zn^2+^ were added in culture medium from DIV1.

HEK293T cells and neurons were all grown at 37°C, 5% CO_2_ in a cell incubator (Thermo).

All animal procedures were carried out in accordance with the animal use rules of South-Central Minzu University and the requisite approvals of the animal use committee.

### Immunostaining

Neurons were fixed in 4% paraformaldehyde at DIV13 and permeabilized with 0.2% Triton X-100, incubated with anti-synapsin1 (rabbit polyclonal antibody), and anti-MAP2 (Microtubule-associated protein 2, mouse monoclonal, Sigma M4403) primary antibodies in PBS with 5% BSA, washed, and visualized using Alexa Fluor-488 goat anti-rabbit and Alexa Fluor-546 goat anti-mouse secondary antibodies (Molecular Probes). Primary antibodies anti-synapsin1 were used to mark synapse of the cultured neuron. The antibodies anti-MAP2 were used to mark the dendrites of cultured neurons. Primary antibodies anti-vGult-1 were used to mark excitatory synapse and anti-GAD65 was used to mark inhibitory synapse. The antibodies anti-tubulin was used to mark the cultured neurons. Images were captured by a Nikon C2 confocal microscope equipped with a 60× oil-immersion objective. Synapse size and number were statistical by ImageJ (NIH).

### Electrophysiological Recordings

Electrophysiological recordings were performed in whole-cell patch clamping mode as described previously (Gong et al., 2016). Micropipettes were pulled from borosilicate glass capillary tubes (World Precision Instruments, Inc.) by using a P-97 micropipette puller (Sutter Instruments). The micropipette solution for recording mEPSC contained 140 mM CsCl, 5 mM NaCl, 10 mM HEPES, 5 mM EGTA, 0.3 mM Na2GTP, and 3 mM Mg2ATP. Adjusted pH to 7.2–7.4 with CsOH. Adjusted osmotic pressure to about 305 mOsm with dd H_2_O. For recording eEPSC, add 5 mM QX-314 to the micropipette solution before use. The bath solution contained 150 mM NaCl, 4 mM KCl, 1 mM MgCl_2_, 2 mM CaCl_2_, 10 mM HEPES, and 10 mM glucose. Adjusted pH of bath solution to 7.2–7.4 with NaOH and adjust osmotic pressure to about 315 mOsm with dd H_2_O. For mEPSC recording, 50 μM AP-5, 100 μM picrotoxin, and 1 μM tetrodotoxin were added to the bath solution before use. Add 50 μM AP-5 and 100 μM picrotoxin to the bath solution for eEPSC recording.

Synaptic currents were monitored with an EPC10 amplifier (HEKA). Miniature events were analyzed in Clampfit 10 (Molecular Devices) using the template matching search function. Single extracellular stimulus pulses (90 μA, 1 ms) were controlled with a Model 2100 Isolated Pulse Stimulator (A-M Systems, Inc.) for eEPSCs measurements. All data were analyzed in Clampfit 10 (Molecular Devices).

### Microarray Hybridization

Total RNA was isolated from cortical neurons cultured with or without 50 μM Zn^2+^ by using TRIzol extraction. RNA was quantified by the NanoDrop ND-2000 (Thermo Scientific) and the RNA integrity was assessed using Agilent Bioanalyzer 2100 (Agilent Technologies). The sample labeling, microarray hybridization, and washing were performed based on the manufacturer’s standard protocols. Briefly, total RNA was transcribed to double strand cDNA, then synthesized into cRNA and labeled with Cyanine-3-CTP. The labeled cRNAs were hybridized onto the microarray. After washing, the arrays were scanned by the Agilent Scanner G2505C (Agilent Technologies).

Feature Extraction software (version10.7.1.1, Agilent Technologies) was used to analyze array images to get raw data. Genespring (version 13.1, Agilent Technologies) was employed to finish the basic analysis with the raw data. To begin with, the raw data was normalized with the quantile algorithm. The probes that at least 100% of the values in any one out of all conditions have flags in “Detected” were chosen for further data analysis. Differentially expressed genes were then identified through fold change as well as P value calculated with a *t*-test. The threshold set for differentially expressed genes was a fold change ≥2.0 and a *P* value ≤0.05. Afterward, GO analysis and KEGG analyses were applied to determine the roles of these differentially expressed mRNAs.

### Plasmid Construction

Full length PTPRM of mice was inserted into the BamHI/EcoRI sites downstream of the UbC promoter of L309 vector. The PTPRM gene was synthesized by GeneCreate.

### Lentiviruses Preparation

Lentiviral expression plasmids and three helper plasmids (pRSV-REV, pMDLg/pRRE, and pVSVG) were co-transfected into HEK293T cells. The transfections were carried out using the polyethylenimine (PEI) method (Jiang et al., 2015). Each well of 6-well-plate including 1 μg pRSV-REV, 1 μg pMDLg/pRRE, 0.5 μg pVSVG, 1.5 μg lentiviral expression plasmids, 12 μl PEI, and 100 μLopti-MEM (Gibco). The virus-containing medium was harvested 48 h after transfection and subsequently concentrated with a sucrose-containing buffer (50 mM Tris-HCl pH 7.4, 100 mM NaCl, 0.5 mM EDTA) at a 4:1 (v/v) ratio and centrifuged at 4°C, 10,000 *g* for 4 h. After centrifugation, the supernatant was carefully removed. PBS buffer was added to the tube for virus resuspension. Then the tube was placed in the 4°C fridge for recovery overnight. All steps were performed under level II biosafety conditions. Neurons were infected with lentiviruses at DIV 6–7 and electrophysiological or immunostaining analyzed at DIV 13–14.

### Real-Time Fluorescence Quantitative PCR Detection

The neuron was collected, subsequently subjected to RNA extraction by adding TRIzol RNA isolation. Reverse transcription was conducted with reverse-transcription kit (Trans Gen, AT311). qPCR was conducted by 2×TSING KE Master qPCR Mix.The results of this study were analyzed with β-actin as the reference gene. The relative expression of the target gene was calculated by 2^–ΔΔ^.

### Statistical Analysis

Prism 6.01 (GraphPad) was used for statistical tests, all of which are described in figure legends.

## Results

### Higher Concentration of Zn^2+^ Damages the Neuronal Synapse Formation

**Figure 1:**
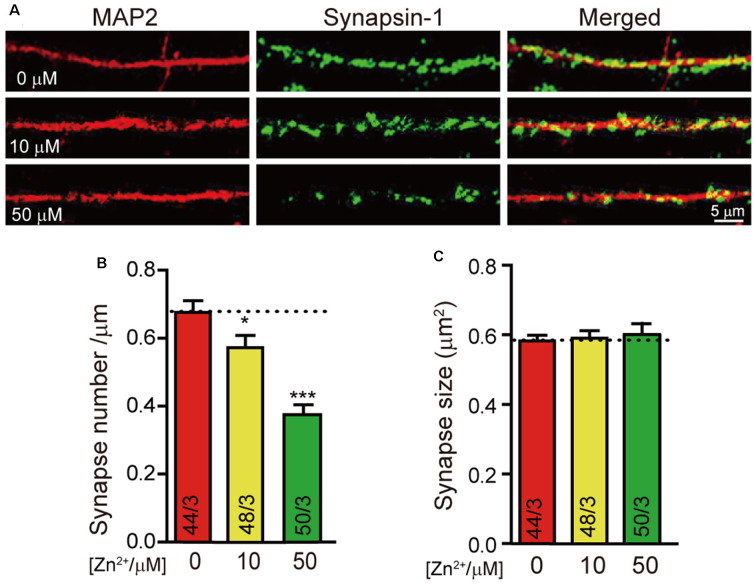
Higher concentration of Zn^2+^ decreases the synapse formation of cultured neurons. **(A)** Representative images of neurons cultured with 0 μM/10 μM/50 μM Zn^2+^ were fixed and labeled by synapsin1 (to mark synapses) and MAP2 antibodies (to mark dendrites). The scale bar in the right lower corner applies to all images. **(B,C)** Summary graphs of synapse number (the number of synapsin1-specific puncta) and synapse size (the area of synapsin1-specific puncta) for all conditions as described in **(A)**. Data information: numbers of cells/independent cultures analyzed are listed in the bars. Data shown in summary graphs are mean values ± SEM. Statistical significance was analyzed by Student’s *t*-test. *p < 0.05; ***p < 0.001. Scale bar: 5 μm.

To investigate the effect of zinc ions on synaptic development, we cultured neurons from newborn mice with the addition of 0 μM, 10 μM, and 50 μM zinc ions to the culture medium. We stained neurons with a universal synapse markersynapsin1 to mark synapse and MAP2 to mark dendrites (Figure 1A) to selectively visualize formed synapses.Quantitation of synapsin1-puncta number as synapse number demonstrated a significant decrease in neurons cultured with zinc ion (Figure 1B). Particularly, the number of formed synapses decreased by about 15% with the addition of 10 μM zinc ion. When 50 μM zinc ions were added, nearly 50% of formed synapses were lost. That is to say, with the higher concentration of zinc ion, the fewer neuronal synapses formed. Besides, we also calculated the synapsin1 puncta size as the synapse size. Statistical results show no relative difference in synapse size in neurons cultured with different concentrations of zinc ion (Figure 1C). These results indicated that synapse formation in cortical neurons is regulated by zinc ion. With the increase of zinc concentration, synaptogenesis of neurons is damaged.

Furthermore, to figure out the effect of zinc ions on excitatory and inhibitory synapse formation, we stained neurons with vGlut-1 and GAD65 to mark excitatory and inhibitory synapse individually (Supplementary Figure S1). We calculated the vGlut1-puncta number and the GAD65 puncta number as the excitatory and inhibitory synapse number. Our results illustrated that the number of excitatory and inhibitory synapses were both reduced with the application of 50 μM zinc ion. The synapse size shows no relative difference in neurons cultured with or without zinc ions.

### The Excitatory Synaptic Transmission Decreases With Higher Concentration of Zn^2+^

**Figure 2:**
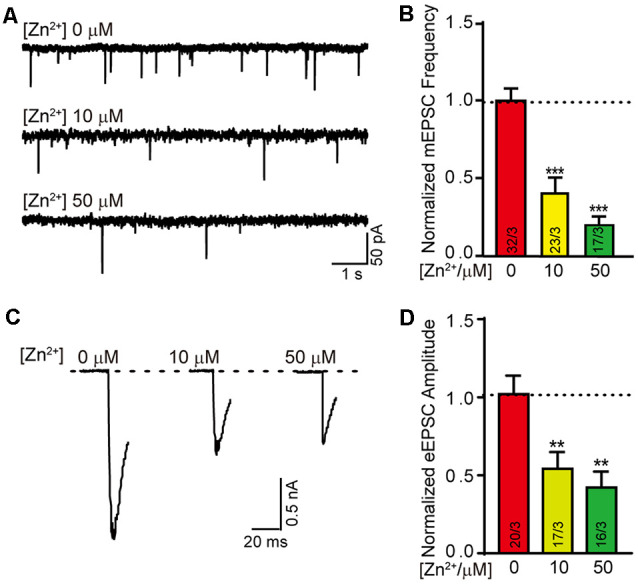
Higher concentration of Zn^2+^ damages the excitatory synaptic transmission. **(A)** Sample traces of mEPSCs recorded in cortical neurons cultured with 0 μM/10 μM/50 μM Zn^2+^. **(B)** Summary graphs of the normalized frequency of mEPSCs for all conditions as described for **(A)**. **(C)** Sample trace of action potential eEPSCs recorded from cortical neurons cultured with 0 μM/10 μM/50 μM Zn^2+^. **(D)** Summary graphs of the normalized amplitude of eEPSCs recorded in the neurons described in panel **(C)**. Data information: numbers of cells/independent cultures analyzed are listed in the bars. Data shown in summary graphs are mean values ± SEM. Statistical significance was analyzed by Student’s *t*-test. **p < 0.01; ***p < 0.001.

Neurotransmitter’s release and reception are the basis of nerve signal transduction. The formation of synapses is closely related to the signal transmission ability of neurons. We next wondered whether the neural signal transduction is affected by zinc ions as well. To test the effect of zinc ion on synaptic transmission, neurotransmitter release was evaluated in neuron cultured with different zinc ion.We systematically measured excitatory mini spontaneous transmitters release and action potential evoked excitatory transmitters release in neurons cultured with 0 μM, 10 μM, and 50 μM zinc ion at DIV 13 respectively (Figures 2A,C). Consistently, the normalized frequency of mini excitatory post synaptic currents (mEPSCs) decreased nearly 60% with the addition of 10 μM zinc ions, and reduced by approximately 80% with the addition of 50 μM zinc ions (Figure 2B). These data suggested that the excitatory spontaneous release decreased with the increase of zinc ion concentration. Furthermore, we observed a concomitant decrease in the amplitude of action potential evoked excitatory postsynaptic current (eEPSC) with the addition of zinc ions. Similarly, the normalized amplitude of eEPSC was reduced by nearly 50% in the presence of 10 μM zinc ions, and reduced by practically 60% at the condition of 50 μM zinc ions (Figures 2C,D). These changes in electrophysiology suggested that neurotransmitters release the ability of neurons impaired with the increase of zinc ion. Taken together, results shown above indicate an expected consequence thatzinc ion changes the ability of synaptic formation and transmission.

### Screening Differentially Expressed Genes Mediated by Zinc Ion With Transcriptome Microarray

**Figure 3:**
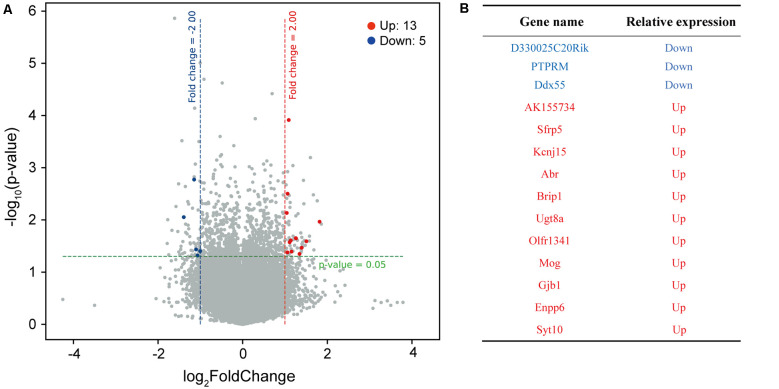
The DEGs of neuron cultured with or without Zn^2+^ analysis by microarray hybridization. **(A)** The volcano map of differential expressed gene. Take neurons cultured with 0 μM as a control group, analyze differentially expressed genes of neurons cultured with 50 μM. The red dots on the map represent significantly upregulated expressed genes, blue dots represent significantly downregulated expressed genes, and black dots represent genes whose expression levels have not changed significantly. The filter condition for differentially expressed genes was a fold change ≥2.0 and a *P* value ≤0.05. **(B)** The table shows the names of differentially expressed genes that encode proteins. Red is upregulated, and blue is downregulated.

The above results suggest that zinc ions can regulate the formation and function of neuronal synapses. To clarify the mechanism, cultured mice cortical neurons were divided into two groups, one group cultured without Zn^2+^, the other group cultured with 50 μM Zn^2+^ addition. Neurons were collected and lysed at DIV13. The mRNA of neurons was extracted, and neuronal transcriptome was analyzed with Agilent sure print G3 mouse Ge v2.0 chip.The differential expression genes were obtained from the two groups’ microarray data. Thirteen upregulated and five downregulated DEGs (differentially expressed genes) were determined, based on the screening conditions of Fc ≥ 2 and P-value (*P* ≤ 0.05), in neurons with 50 μM Zn^2+^ (Figure 3A). Among the 18 DEGs, four of them are link RNA. The remaining 14 gene names are shown in Figure 3B. Next, the 14 DEGs were pooled and a heat map was made according to the expression amount (Supplementary Figure S2). The heat map is used to reveal the differences of gene expression patterns from different samples. The gene expression patterns of different samples are closer, the clustering relationship is more similar. It can be seen that the differential gene expression patterns are more similar in the experimental group (50 μM Zn^2+^) or in the control group (0 μM Zn^2+^), which is in line with the law of repeated samples. This illustrated the correctness of sampling and data analysis. Among the DEGs (Figure 3B), we noticed that the PTPRM is involved in cell recognition, signal transduction, or cell proliferation and differentiation. Therefore, we hypothesized that PTPRM is associated with synapse formation during neuronal development.

Furthermore, we analyzed the mRNA expression of other genes in the PTPRM family (Supplementary Figure S3). The results showed that only the expression of PTPRd decreased after adding zinc ions to the culture medium, whereas the expression of the other members of PTPR family was unaltered. Although the decrease was significant, the gene PTPRd was not included in our DEGs list because the fold change did not reach 2.

### PTPRM Is Critical for Synapse Formation Mediated by Zn^2+^

**Figure 4:**
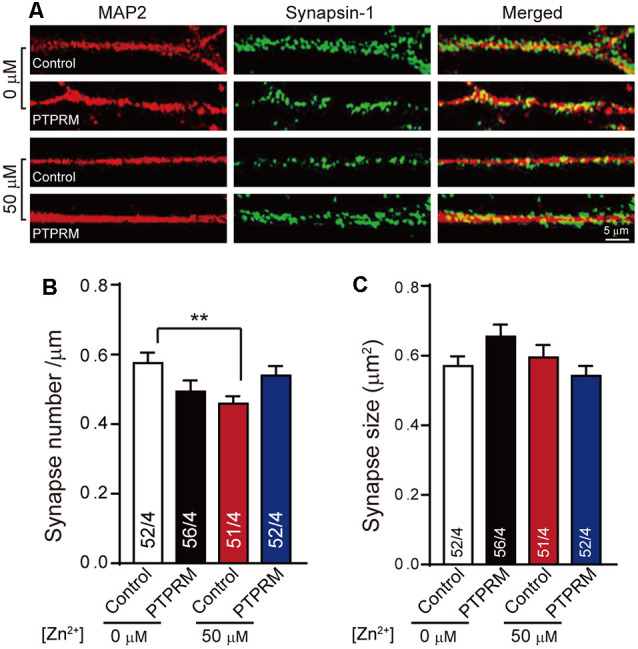
PTPRM is critical for synapse formation regulated by Zn^2+^. **(A)** Representative images of neurons overexpressed PTPRM or not cultured with/without 50 μM Zn^2+^ when expressed PTPRM or not. Neurons were fixed and labeled by synapsin1 (to mark synapses) and Map AP2 (to mark dendrites) antibodies. The scale bar in the right lower corner applies to all images. **(B)** Summary graphs of synapse number calculated from the number of synapsin1-specific puncta for the conditions described in **(A)**. **(C)** Summary graphs of synapse size calculated from the area of synapsin1-specific puncta for the conditions described in **(A)**. Data information: numbers of cells/independent cultures analyzed are listed in the bars. Data shown in summary graphs are mean values ± SEM. Statistical significance was analyzed by Student’s *t*-test. **p < 0.01. Scale bar: 5 μm.

To validate whether PTPRM is critical for synapse formation, we examined the synapse number and size in PTPRM over expressing neurons cultured with or without Zn^2+^ by immunostaining. We quantified the PTPRM expression by qPCR. The mRNA level of PTPRM was decreased by 70% with the addition of 50 μM zinc ions, which is consistent with the results of chip data. The mRNA level of PTPRM was nearly doubled in virus-infected neurons (Supplementary Figure S4). Like Figure 1, then we stained neurons with anti-synapsin1 and anti-MAP2 to mark synapses and dendrites separately (Figure 4A). Quantitation of synapsin1 puncta number shows that the expression of PTPRM significantly rescued the synapse number decreased caused by Zn^2+^ (Figure 4B). As always, the size of synapses has not change (Figure 4C). These data indicated that PTPRM participates in the process of synapse formation regulated by Zn^2+^.

### PTPRM Plays a Key Role in Excitatory Synaptic Transmission Mediated by Zn^2+^

**Figure 5:**
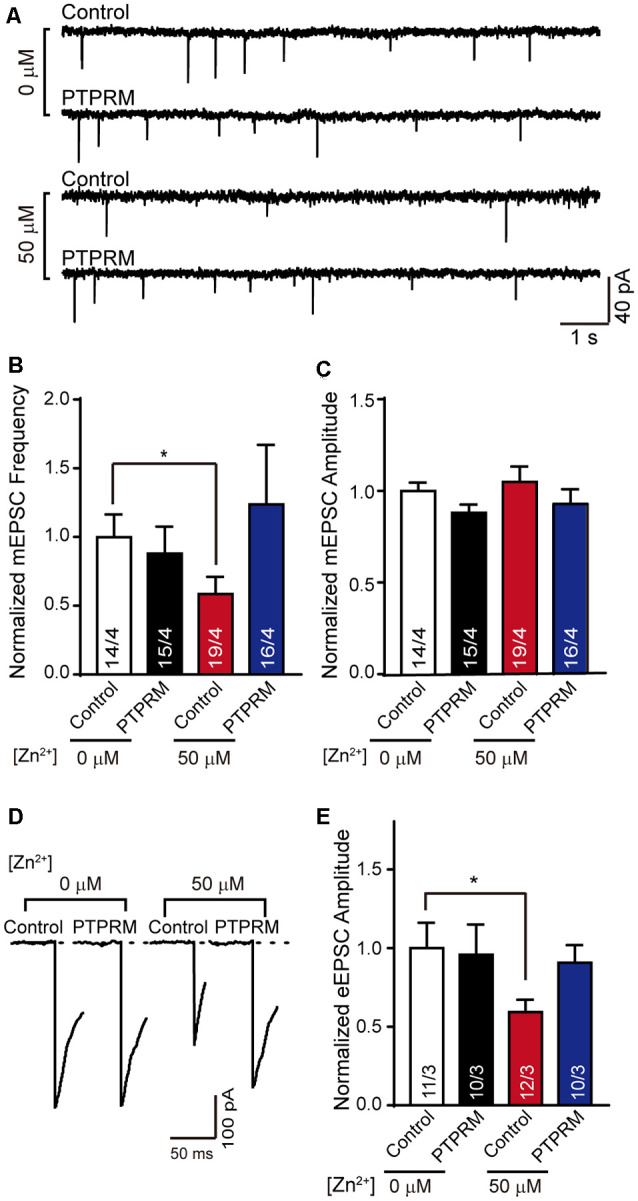
PTPRM is key for excitatory synaptic signal transduction mediated by Zn^2+^. **(A)** Representative traces of mEPSCs recorded in cortical neurons overexpressed PTPRM or not cultured with/without 50 μM Zn^2+^ when expressed PTPRM or not. **(B)** Summary graphs of the normalized frequency of mEPSCs for all conditions as described in panel **(A)**. **(C)** Statistical summary of the normalized amplitude of mEPSCs for all conditions as described in panel **(A)**. **(D)** Representative of eEPSCs recorded from cortical neurons described in panel **(A)**. **(E)** Statistical summary of normalized amplitude of eEPSCs recorded in the neurons described in panel **(A)**. Data information: numbers of cells/independent cultures analyzed are listed in the bars. Data shown in summary graphs are mean values ± SEM. Statistical significance was analyzed by Student’s *t*-test. *p < 0.05.

Changes in synapse formation are often accompanied by the variation in neural signal transmission. Since PTPRM regulated synapse formation in the presence of Zn^2+^, is synapse transmission also regulated by PTPRM? To answer this question, we tested the mEPSC and eEPSC of the cultured neurons with or without PTPRM overexpression and in the presence of 50 μM Zn^2+^or not (Figure 5). Consistent with the data in Figures 2A,B; the normalized frequency of mEPSC was decreased with the addition of 50 μM Zn^2+^ (Figures 5A,B). Moreover, the overexpression of PTPRM rescued the decreased frequency of mEPSC induced by 50 μM Zn^2+^. These results illustrate that the PTPRM is vital for excitatory synaptic transmission mediated by Zn^2+^. The mEPSC amplitude was unaffected in all conditions, suggesting the major presynaptic effect caused by the PTPRM (Figure 5C). Similarly, the normalized amplitude of eEPSC was deceased with the addition of 50 μM Zn^2+^, which is consistent with the data in Figures 2C,D. And the decrease of eEPSC amplitude was rescued with the overexpression of PTPRM (Figures 5D,E). Collectively, these results provide consistent evidence to reveal that PTPRM is indispensable for excitatory synaptic transmission mediated by Zn^2+^.

## Discussion

Previous studies have shown that zinc ions play vital roles in the physiology and pathology of the nervous system, but its regulatory mechanism is still unclear (Sensi et al., 2009; Choi et al., 2020). Synapse is the junction of our brain’s information communication and the basis for the realization of the brain’s advanced functions (Südhof, 2014). However, little is known about whether synapse formation is regulated by zinc ions. In this article, we try to figure out this question.

Firstly, we found that the synapse formation in cultured cortical neurons was decreased with the addition of 50 μM zinc ions (Figure 1). Secondly, the ability of neuronal signal transduction changed with the addition of zinc ions according to the mEPSC and eEPSC data in Figure 2. These data indicated that synapse formation and synaptic transmission were regulated by zinc ions. The synapse marker used here was the antibody of synapsin-1, which can label both excitatory and inhibitory synapses (Gitler et al., 2004). However, we only monitored the synaptic transmission of excitatory synapses (Figures 2, 5). We speculate that inhibitory synapses have similar effects, which needs to be confirmed by further evidence.

For figuring out the mechanism of synapse formation regulated by zinc ions, the gene expression of neurons cultured with or without zinc was analyzed, which provided a basis for further study. After analyzing, we found that 14 genes encoding proteins were differentially expressed. According to the functional description of DEGs, we noticed that the PTPRM plays a role in cell adhesion, suggesting that PTPRM is essential for in-cell growth, activation, and signal transduction (Mukouyama et al., 1995; Barazeghi et al., 2019; Im et al., 2020; Song et al., 2021). Excessive zinc ions lead to a decrease in the expression of PTPRM (Figure 3). Therefore, we suspect that PTPRM is involved in the synaptic formation of zinc ions regulation. To test this hypothesis, we overexpressed PTPRM in neurons, and tested the synapse formation and synaptic function. More interestingly, using electrophysiological and immunofluorescence experiments, we identified PTPRM could recover the defects of synapse formation and transmission caused by zinc ions (Figures 4, 5). These results suggest that PTPRM plays an indispensable role in the synaptic formation regulated by zinc ions. It is inevitable that whether other differentially expressed genes also play a key role in synaptic formation regulated by zinc ions remains unknown.

However, how PTPRM mediates synapse formation regulated by zinc ions remains unclear. The protein expressed by PTPRM is receptor-type tyrosine-protein phosphatase mu, which is a type IIB receptor protein tyrosine phosphatase. PTPRM is a transmembrane protein with a single transmembrane domain. The extracellular region includes one MAM (meprin/A5/m) domain, one immunoglobulin (Ig)-like domain, and four fibronectin (FN) type III repeats, and the intracellular region consists of two conserved phosphatase domains (den Hertog et al., 1999). The membrane-proximal C1 domain is responsible for the catalytic activity of PTPRM. The ectodomain of PTPRM interacts with itself in an anti-parallel homophilic manner, involving cell-cell contact (Brady-Kalnay et al., 1993; Gebbink et al., 1995; Aricescu et al., 2007). The PTPRM associates with and appears to mediate axon outgrowth-promoting effects through interactions with cadherins (Brady-Kalnay et al., 1998). Downregulation of PTPRM expression can decrease retinal ganglion cell axon outgrowth on an N-cadherin substrate (Burden-Gulley and Brady-Kalnay, 1999). This demonstrates that PTPRM is a key regulator of neurite outgrowth. These studies suggest that the synapse formation of cortical neurons may be regulated by PTPRM, probably because PTPRM regulates the development of axons. Of course, there are reports showing that LAR-RPTPs (the other sub family of PTPRs) are also synaptic adhesion molecules that form trans-synaptic adhesion complexes by binding to various postsynaptic adhesion ligands, such as Slit-, Slitrks, and IL1RAPL1 (Won et al., 2017; Won and Kim, 2018). This enlightens us that PTPRM may also participate in synaptic formation as a synaptic adhesion molecule. Then zinc ions may indirectly regulate synapse formation by regulating the expression of PTPRM. To figure out how PTPRM mediates synapse formation regulated by zinc ions will be one direction of future exploration.

In addition, the function of other DEGs such as Sfrp5 or Abr regulated by zinc ions is unclear. Do they play a role in synapse formation or in other biological functions of neurons? How are they incorporated with PTPRM? More experimental evidence is needed.

Zinc is one of the most abundant nutritionally essential elements in our body. In particular, zinc is a biological component that plays an important physiological role in the central nervous system, and a pathophysiological role in major neurological disorders. In this article, we identified PTPRM is involved in Zn^2+^ related neuronal synaptic function, hoping to provide new ideas for understanding the regulation of brain function by zinc ions.

## Data Availability Statement

The original contributions presented in the study are included in the article/Supplementary Material, further inquiries can be directed to the corresponding author/s.

## Ethics Statement

The animal study was reviewed and approved by The animal use committee of South-Central Minzu University.

## Author Contributions

ML and YM performed the experiments. XM, ML, HC, and HM analyzed data. ML, YM, and JG prepared the figures. XY and JL designed the research. JG and XY wrote the main manuscript text. All authors contributed to the article and approved the submitted version.

## Funding

This research was supported by the National Science Foundation of China (32170699) and National Science Foundation of Hubei (2020CFA025).

## Conflict of Interest

YM was employed by the company Wuhan Institute of Biological Products. The remaining authors declare that the research was conducted in the absence of any commercial or financial relationships that could be construed as a potential conflict of interest.

## Publisher’s Note

All claims expressed in this article are solely those of the authors and do not necessarily represent those of their affiliated organizations, or those of the publisher, the editors and the reviewers. Any product that may be evaluated in this article, or claim that may be made by its manufacturer, is not guaranteed or endorsed by the publisher.

## Supplementary Materials

Supplementary Figure S1 50 μMZn^2+^ decrease both the excitatory and inhibitory synapse formation. **(A)** Representative images of neurons cultured with 0 μM or 50 μM Zn^2+^ were fixed and labeled by vGult-1 (to mark excitatory synapses) and Tubulin antibodies (to mark neurons). The scale bar in the right lower corner applies to all images. **(B,C)** Summary graphs of excitatory synapse number (the number of vGult-1-specific puncta) and synapse size (the area of vGult-1-specific puncta) for all conditions as described for **(A)**. **(D)** Representative images of neurons cultured with 0 μM or 50 μM Zn^2+^ were fixed and labeled by GAD65 (to mark inhibitory synapses) and Map-2 antibodies (to mark dendrites). The scale bar in the right lower corner applies to all images. **(E,F)** Summary graphs of inhibitory synapse number (the number of GAD65-specific puncta) and synapse size (the area of GAD65-specific puncta) for all conditions as described for **(D)**. Data information: numbers of neurons analyzed are at least 13. Data shown in summary graphs are mean values ± SEM. Statistical significance was analyzed by Student’s *t*-test. ***p* < 0.01. Scale bar: 5 μm.

Supplementary Figure S2 The heat map of differentially expressed gene. A heat map was made based on the amount of expression. The horizontal is the sample, and the vertical are the differentially expressed genes.

Supplementary Figure S3 The relative mRNA expression of the genes of PTPR family. The mRNA expression profiles of PTPR family determined by chip data from neurons cultured with or without Zn^2+^. Data shown in summary graphs are mean values ± SEM. Statistical significance was analyzed by Student’s *t*-test. **p* < 0.05.

Supplementary Figure S4 The relative mRNA expression of PTPRM determined by qPCR. The mRNA expression was quantitative analyzed from the neurons cultured with or without 50 μM zinc ions, infected with viruses expressing control vector or PTPRM. β-actin was used as the reference gene. Data shown in summary graphs are mean values ± SD, *n* = 3. Statistical significance was analyzed by Student’s *t*-test. **p* < 0.05; ***p* < 0.01; ****p* < 0.001.
