# Supplementary figures and images for: PTPRM Is Critical for Synapse Formation Regulated by Zinc Ion
Source: Front Mol Neurosci. 2022 Mar 21;15:822458. doi: 10.3389/fnmol.2022.822458 (PMC8977644; doi:10.3389/fnmol.2022.822458)

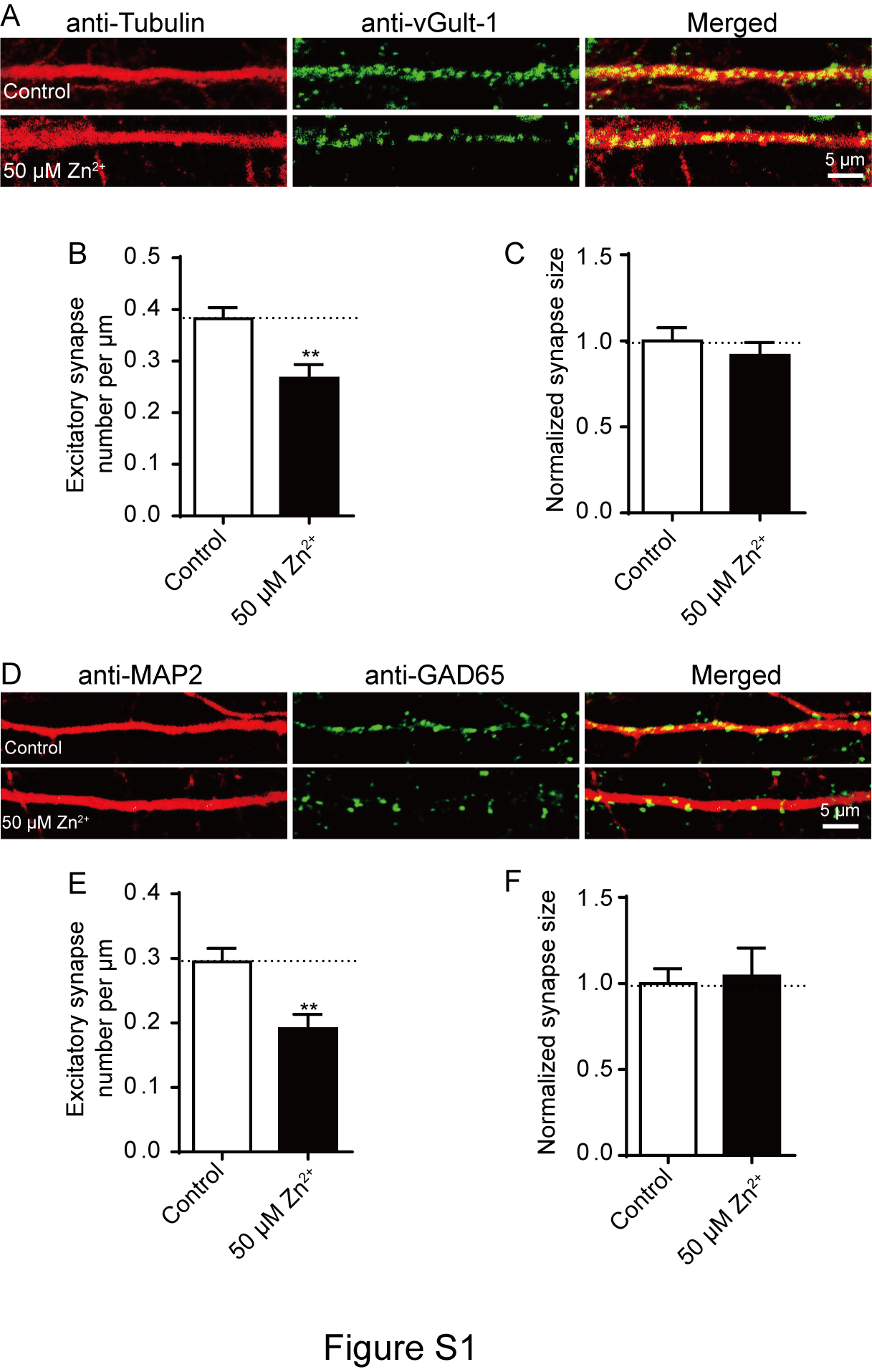

Supplement: Supplementary Figure S1 — 50 μMZn2+ decrease both the excitatory and inhibitory synapse formation. (A) Representative images of neurons cultured with 0 μM or 50 μM Zn2+ were fixed and labeled by vGult-1 (to mark excitatory synapses) and Tubulin antibodies (to mark neurons). The scale bar in the right lower corner applies to all images. (B,C) Summary graphs of excitatory synapse number (the number of vGult-1-specific puncta) and synapse size (the area of vGult-1-specific puncta) for all conditions as described for (A). (D) Representative images of neurons cultured with 0 μM or 50 μM Zn2+ were fixed and labeled by GAD65 (to mark inhibitory synapses) and Map-2 antibodies (to mark dendrites). The scale bar in the right lower corner applies to all images. (E,F) Summary graphs of inhibitory synapse number (the number of GAD65-specific puncta) and synapse size (the area of GAD65-specific puncta) for all conditions as described for (D). Data information: numbers of neurons analyzed are at least 13. Data shown in summary graphs are mean values ± SEM. Statistical significance was analyzed by Student’s t-test. **p < 0.01. Scale bar: 5 μm. [file Image_1.TIF]

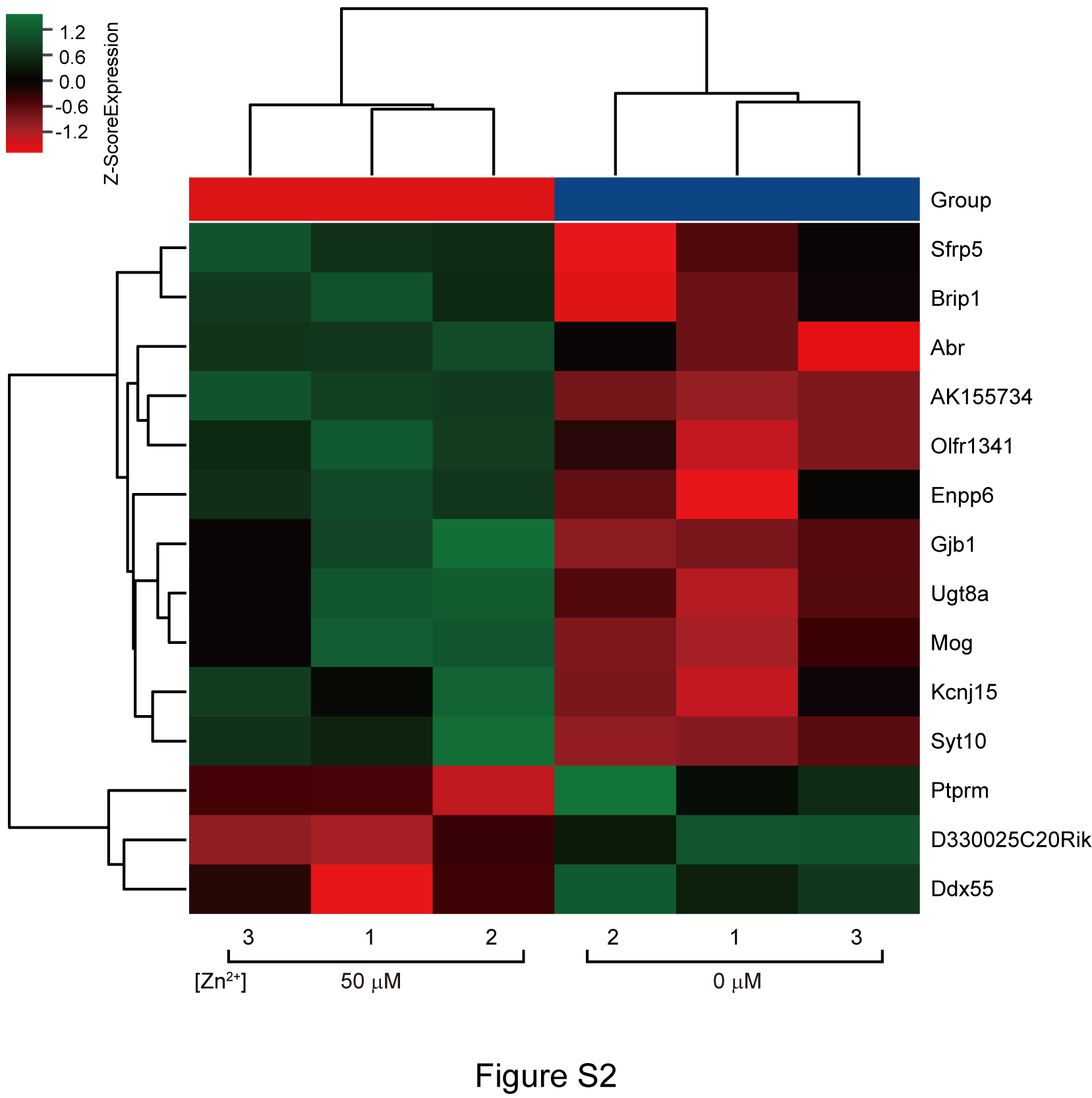

Supplement: Supplementary Figure S2 — The heat map of differentially expressed gene. A heat map was made based on the amount of expression. The horizontal is the sample, and the vertical are the differentially expressed genes. [file Image_2.TIF]

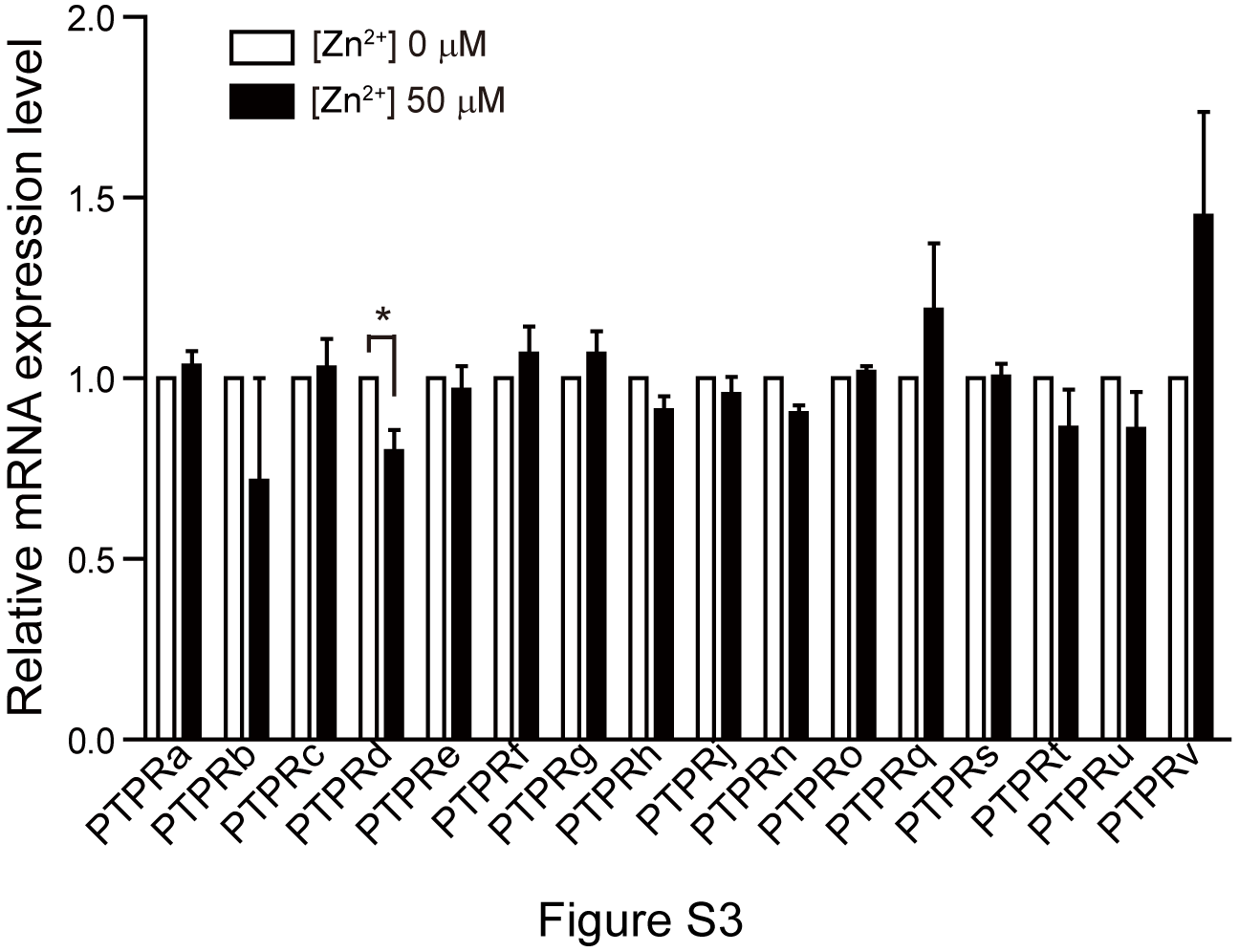

Supplement: Supplementary Figure S3 — The relative mRNA expression of the genes of PTPR family. The mRNA expression profiles of PTPR family determined by chip data from neurons cultured with or without Zn2+. Data shown in summary graphs are mean values ± SEM. Statistical significance was analyzed by Student’s t-test. *p < 0.05. [file Image_3.TIF]

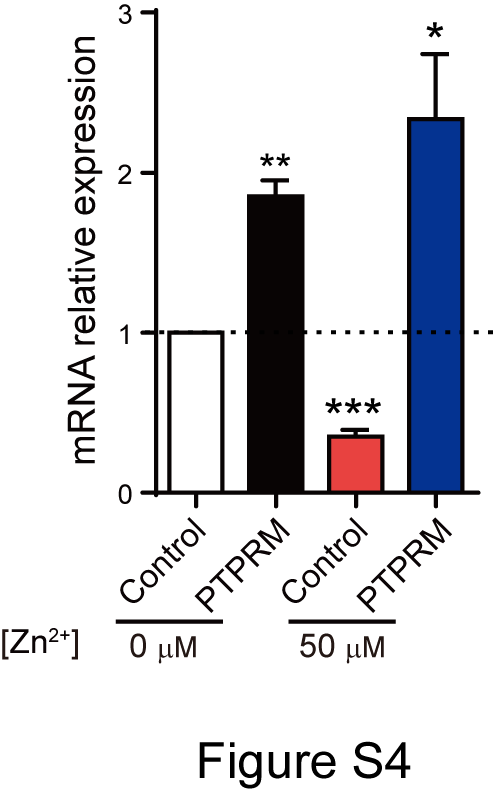

Supplement: Supplementary Figure S4 — The relative mRNA expression of PTPRM determined by qPCR. The mRNA expression was quantitative analyzed from the neurons cultured with or without 50 μM zinc ions, infected with viruses expressing control vector or PTPRM. β-actin was used as the reference gene. Data shown in summary graphs are mean values ± SD, n = 3. Statistical significance was analyzed by Student’s t-test. *p < 0.05; **p < 0.01; ***p < 0.001. [file Image_4.TIF]
